# Novel insights into the role of immunomodulatory extracellular vesicles in the pathogenesis of liver fibrosis

**DOI:** 10.1186/s40364-024-00669-8

**Published:** 2024-10-12

**Authors:** Jiaxuan Li, Yue Yuan, Qinggang Fu, Min Chen, Huifang Liang, Xiaoping Chen, Xin Long, Bixiang Zhang, Jianping Zhao, Qian Chen

**Affiliations:** 1 Division of Gastroenterology, Department of Internal Medicine at Tongji Hospital, Tongji Medical College, Huazhong University of Science and Technology, Wuhan, 430030 China; 2 Hepatic Surgery Center, Tongji Hospital, Tongji Medical College, Huazhong University of Science and Technology, Wuhan, 430030 China; 3 Hubei Key Laboratory of Hepato-Pancreato-Biliary Diseases, Wuhan, 430030 China

**Keywords:** Extracellular vesicles, Inflammatory microenvironment, Liver fibrosis

## Abstract

Liver fibrosis, a chronic and long-term disease, can develop into hepatocellular carcinoma (HCC) and ultimately lead to liver failure. Early diagnosis and effective treatment still face significant challenges. Liver inflammation leads to liver fibrosis through continuous activation of hepatic stellate cells (HSCs) and the accumulation of immune cells. Intracellular communication among various immune cells is important for mediating the inflammatory response during fibrogenesis. Extracellular vesicles (EVs), which are lipid bilayer membrane-enclosed particles naturally secreted by cells, make great contributions to cell-cell communication and the transport of bioactive molecules. Nearly all the cells that participate in liver fibrosis release EVs loaded with lipids, proteins, and nucleic acids. EVs from hepatocytes, immune cells and stem cells are involved in mediating the inflammatory microenvironment of liver fibrosis. Recently, an increasing number of extracellular vesicle-based clinical applications have emerged, providing promising cell-free diagnostic and therapeutic tools for liver fibrosis because of their crucial role in immunomodulation during pathogenesis. The advantages of extracellular vesicle-based therapies include stability, biocompatibility, low cytotoxicity, and minimal immunogenicity, which highlight their great potential for drug delivery and specific treatments for liver fibrosis. In this review, we summarize the complex biological functions of EVs in the inflammatory response in the pathogenesis of liver fibrosis and evaluate the potential of EVs in the diagnosis and treatment of liver fibrosis.

## Introduction

Liver fibrosis is the terminal phase of various chronic liver diseases, including viral hepatitis, metabolic dysfunction-associated steatohepatitis (MASH), alcoholic liver disease (ALD), and gene-related liver diseases [1]. Liver fibrosis can progress to cirrhosis, which is the eleventh most common cause of death and accounts for 3.5% of all deaths globally [2]. The pathogenesis of liver fibrosis involves long-term activation of hepatic stellate cells (HSCs), proliferation of profibrogenic cells, continuous inflammatory response, and excessive extracellular matrix decomposition. Once liver injury occurs, quiescent HSCs differentiate into activated myofibroblasts, which secrete proinflammatory cytokines and chemokines to attract immune cells infiltrating the injured site, thus inducing persistent inflammation [3, 4]. In contrast, signals from the extracellular matrix, injured tissues, and infiltrating immune cells perpetuate the activation of HSCs, which create a chronic cycle of inflammation and fibrous scar formation [4]. Although inflammation is mostly a physiological and rewarding process in response to liver injury, a sustained and uncontrolled inflammatory response leads to irreversible damage, resulting in liver fibrosis.

However, the minimal information for studies of extracellular vesicles 2018 (MISEV 2018) guidelines define the natural particles derived from cells as extracellular vesicles (EVs), especially in the light of the widespread use of the term “exosome” and “microvesicle” over the past decades [5]. Historically, the term “exosome” represented small EVs (30–100 nm) generated by reticulocytes previously. Today, exosomes are understood to form through the invagination of endosomal membrane and are released through multivesicular bodies (MVBs) fusion to plasma membrane [6]. EVs were initially considered a way for cells to expel waste and maintain cellular homeostasis or for cancer cells to facilitate tumor progression subsequent metastasis [7]. Increasingly, scientists have recognized the essential role of EVs in cell-cell communication, as they deliver specific cargos such as lipids, proteins, RNAs, metabolites, and enzymes [8]. EVs have been confirmed to have a significant value in immune responses, viral infection, cardiovascular diseases, nervous degenerative diseases, and cancers [8, 9]. Additionally, previous studies have demonstrated that EVs derived from immune cells or immune-related cells exert various and complex effects on chronic liver diseases, mediating the progression of liver fibrosis [10]. Therefore, determining the mechanism and precise effects of EVs in the inflammatory microenvironment of liver fibrosis is necessary, and efforts are needed to explore novel directions for understanding the underlying mechanisms of liver fibrosis and improve targeted therapies.

The cargo of EVs varies with the etiology and severity of liver fibrosis, which suggests their diagnostic and prognostic potential in evaluating liver fibrosis [11]. Compared to the widely used “gold standard” for diagnosing liver fibrosis, EV-based measures are cell-free, non-invasive, safe, and easily accessible alternative. However, their diagnostic specificity and sensitivity still require improvement before being fully integrated into clinical use [12]. The therapeutic value of exosomes in liver fibrosis and chronic liver diseases has been investigated in both animal models and in vitro experiments [13]. EVs function as therapeutic agents in several ways: (a) they work as drug delivery platforms to protect their cargo from degradation and clearance [14]; (b) modified EVs enhance the target specificity and the therapeutic efficacy of therapeutic EVs [15]; and (c) the administration of some natural EVs produced by immune cells or mesenchymal stem cells (MSCs) has been shown to alleviate liver fibrosis through immunomodulation [16]. As biogenic natural transporters, EVs exhibit low immunogenicity and oncogenicity. Moreover, they also have excellent permeability to penetrate various biological barriers compared to synthetic nanovesicles and have stable structures in plasma [17]. However, EV-based therapies are lacking in clinical trials and mature technologies. This review briefly discusses the role of EVs in the immunomodulation of liver fibrosis, as well as their advantages and limitations in clinical applications.

## EVs

### Structure and composition of EVs

EVs are lipid bilayer membrane-enclosed nanoparticles secreted by various cells [5]. According to MISEV2018, EVs can be divided into small EVs (< 100–200 nm), large EVs, and/or medium EVs (> 200 nm). In addition to physical properties, other classification criteria for EV subtypes include biological components, culture conditions, or the origination of EV-producing cells [5]. For example, EVs can be categorized as CD63+/CD81+- EVs and Annexin A5-stained EVs based on their biological composition. Furthermore, EVs are also classified by their cellular origin, such as those derived from normal, hypoxia, and tumor apoptotic cells [5, 18]. Currently, EVs are a type of well-studied ‘deliverymen’ with various biological activities that can transfer proteins, lipids, cytokines, and nucleic acids, including mRNAs, long noncoding RNAs, and microRNAs (miRNAs). They can be secreted by nearly all cells and are widely distributed in body fluids, such as plasma, saliva, cerebral spinal fluid, urine, and gastric acid [19].

In addition to their cargo, EVs are composed of a double-layered lipid membrane and EV surface proteins, which can be classified into public and specific components [18]. Public components represent proteins involved in EV biogenesis and secretion, including tetraspanins (such as CD63, CD81, TSPAN6, TSPAN8, Flotilin1, and Flotilin2), endosomal-sorting complex is required for transport (ESCRT), complex-related proteins such as Tumor Susceptibility Gene 101 (TSG101) and ALG-2 interacting protein X (ALIX) and heat shock proteins, including HSP60, 70, 90 [6, 18]. Some of these proteins serve as markers for detecting and characterizing EVs. In contrast, the effects of cell-specific proteins [including Major Compatibility Complex I (MHC I), C-X-C motif chemokine receptor 4 (CXCR4), and CD86] are dependent mainly on their donor cells or unique functions, highlighting the heterogeneity of EVs [6, 18]. In addition, lipids in the EV membrane generally include phospholipids, cholesterol, ganglioside GM3, and sphingomyelin. Nevertheless, the percentage of these lipids in EVs is related to the type of donor cell and the cellular requirements [20].

### Biogenesis of EVs

The process of EV biogenesis is intricate. Exosomes and microvesicles, which are involved in primary EV subtypes, both require membrane-trafficking processes but differ in the means of biogenesis [6]. In exosomes, cell membranes invaginate and form early sorting endosomes (ESEs) via the trans-Golgi network and endoplasmic reticulum [9, 21]. Next, ESEs mature into late-sorting endosomes (LSEs) and are finally compressed into MVBs, which can be disintegrated by lysosomes in the degradative pathway, or they can merge into the plasma membrane and secrete exosomes in the secretory pathway [22, 23]. In addition, intraluminal vesicles (ILVs), which are precursors of exosomes, are formed by endosomal membrane invagination of LSEs and are enclosed by MVBs [21]. Finally, ILVs can fuse with the plasma membrane and are thus released via the exosomal pathway, or they can interact with surface proteins to induce calcium influx [23]. Secreting exosomes enter circulation and are widely transported. In addition, the ability of MVBs to fuse with the plasma membrane is contingent on the concentration of cholesterol. Previous studies have demonstrated that MVBs lack cholesterol and enter the exosomal pathway [18].

**Fig. 1:**
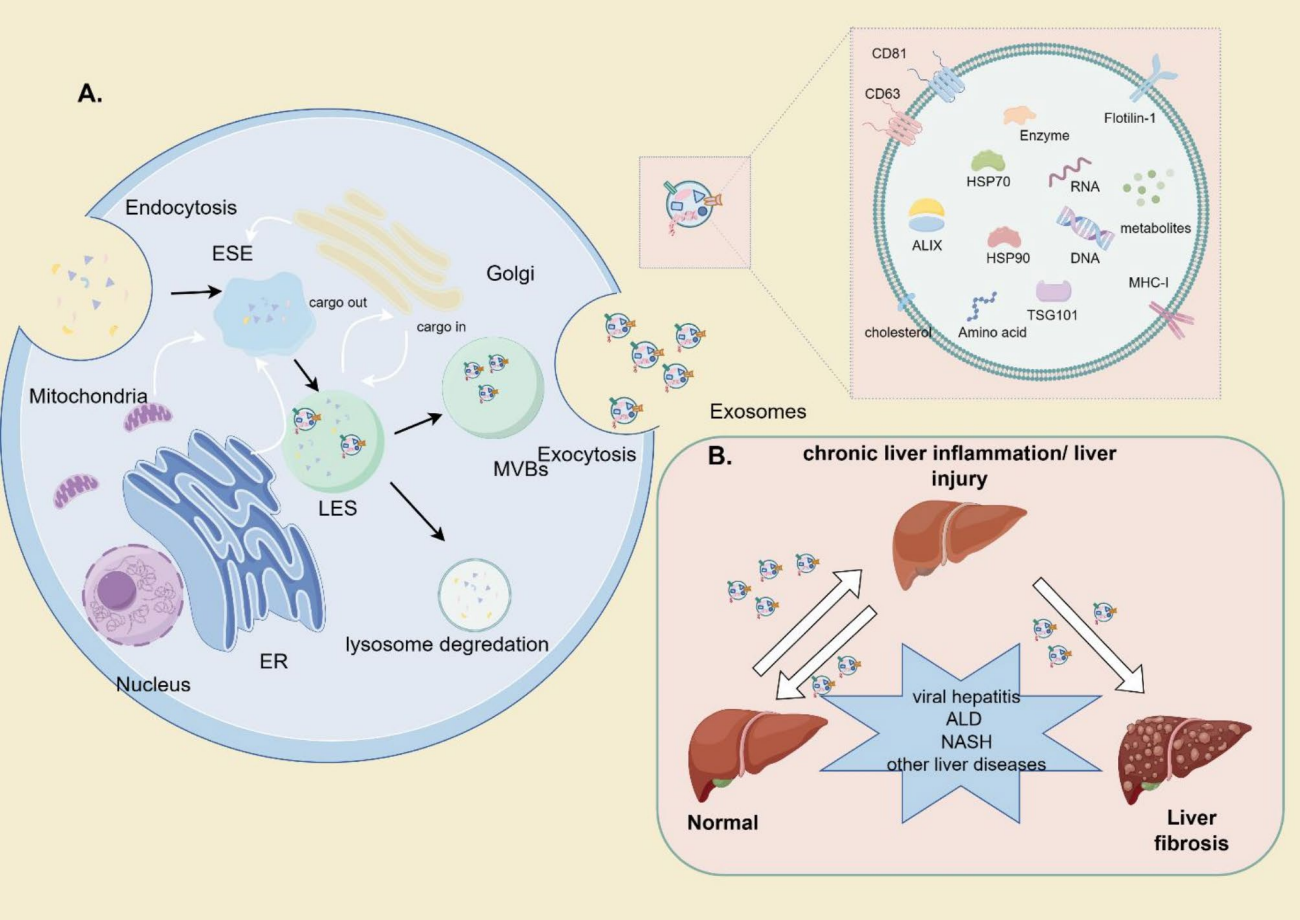
(**A**) Biogenesis and composition of exosomes. ESEs via endocytosis. In addition, ESEs mature into late endosomes (LESs) and form MVBs. Exosomes released by MVBs can be disintegrated by lysosomes or fuse with the plasma membrane. Finally, exosome-producing cells secrete exosomes via exocytosis. Public proteins expressed in exosomes include teraspanins (CD81, CD63), ESCRT-complex related proteins (TSG101, Alix), and heat shock proteins (HSP60,70,90). (**B**) The occurrence of liver fibrosis. Viral hepatitis, ALD, MASH, and other liver diseases could induce chronic liver injury/inflammation, causing liver fibrosis. EVs provide a platform for intercellular communication during the development of hepatic inflammation and liver fibrosis

MVB transport and ILV production are controlled mainly by the ESCRT-dependent pathway and certain accessory proteins, such as ALIX [9]. Nevertheless, an alternative pathway for EV biogenesis does not rely on the ESCRT machinery. Instead, it requires sphingomyelinase in place of ESCRT. Moreover, emerging evidence shows that TSG101, syndecan-1, and soluble N-ethylmaleimide-sensitive factor attachment protein receptor (SNARE) complex proteins also contribute to EV biogenesis and secretion [6, 22]. The roles of these proteins may intersect, and the exact mechanism requires further analysis [9] (Fig. 1A).

Microvesicles, on the other hand, are formed by the outward budding and fission of the plasma membrane which is enriched in cholesterol. These microvesicles are then promptly discharged into the extracellular space [6].

### Biological role of EVs

Releasing cytokines distantly, exchanging information via gap junctions, and interacting with surface proteins are methods of intracellular communication [24]. With growing research on EVs, their essential function as vehicles for transporting genetic information, cytokines, and proteins, which affects intracellular communication, is becoming increasingly evident [25]. Previous studies have shown that EVs transfer their cargo to mediate the biological activities and conditions of recipient cells [6]. In general, EVs loaded with functional cargo affect their targeting cells via several pathways: by directly merging into target cells; mediating their gene expression by loading RNA, lipids, and proteins; and binding to surface proteins via receptor‒ligand interactions, endocytosis, micropinocytosis, and phagocytosis [25, 26]. Conversely, Cargo K et al. noted that cellular requirements influence EV secretion by altering the habitat of reticulocytes and detecting the lipid composition of EVs [27]. Therefore, reciprocal interactions exist between generation and uptake of EVs.

As EVs play critical roles genetic information exchange and active molecule delivery, increasing evidence has confirmed their involvement in various physiological and pathological activities, including cell proliferation, cell differentiation, cell apoptosis, angiogenesis, and intracellular communication [25, 28]. In addition, both adaptive and innate inflammatory responses can be influenced by EVs [9]. They may mediate inflammation by delivering antigen-presenting peptides, increasing inflammasome activation and transferring cargo such as antigens and RNA [20, 23, 29].

## Role of the inflammatory microenvironment in liver fibrogenesis

As a possible outcome of most chronic liver diseases, the etiology of liver fibrosis includes hepatotoxic liver diseases, such as viral hepatitis, alcoholic liver diseases, nonalcoholic steatohepatitis, and autoimmune liver diseases. Additionally, cholestatic injury, including biliary atresia, primary sclerosing cholangitis, and primary biliary cholangitis, also contribute to liver fibrosis [30] (Fig. 1B). Continuous or repetitive liver injury and sustained inflammatory reactions both cause liver fibrosis, which is characterized by excessive extracellular matrix (ECM) accumulation, changes in the type of collagen (collagen IV replaced by collagens I and III), and persistent HSC activation [31].

Liver fibrogenesis begins with damaged hepatocytes, which produce proinflammatory cytokines such as tumor necrosis factor-α (TNF-α), IL-6 and C-C motif chemokine ligand (CCL2) to regulate the liver immune response. These damaged hepatocytes also release high-mobility group box 1 (HMGB1) and IL-33 to activate HSC [4, 32]. Activated HSCs increase the production of ECM and secrete cytokines to recruit leukocytes, with the involvement of damaged hepatocytes [32]. For example, IL-17 from activated HSCs, ATP and formyl peptides from injured hepatocytes induce neutrophils to migrate to the site of injury in the early phase of liver inflammation. Monocytes and macrophages are recruited to damaged sites via CCL2 secreted by injured hepatocytes. These recruited macrophages produce transforming growth factor-β (TGF-β), which activates HSCs and leads to the development of monocyte-derived macrophages (MoMFs) that sustain inflammation [4, 32]. Recruited hepatic macrophages produce TGF-β to continuously stimulate HSCs and activate the NF-κB pathway to maintain the survival of the activated HSCs. In addition, they generate reactive oxygen species (ROS) that promote the production of collagen I, a key intergradient of overproduced ECM in HSCs [33]. TGF-β is the key profibrogenic cytokine in activated HSCs. Besides macrophages, Th 17 cells and neutrophils can secrete IL-17, which increases the expression of the TGF-β pathway. Furthermore, activated HSCs generate TGF-β and maintain its active state in an autocrine manner [4].

Liver inflammation can be considered as a ‘double-edged sword’ in the development of liver fibrosis. Some immune cells in the liver, including macrophages, natural killer (NK) cells, natural killer T (NKT) cells, dendritic cells (DCs), and T lymphocytes, have antifibrotic effects on liver fibrogenesis [30]. Apoptosis and inactivation of activated hepatic stellate cells (aHSCs), regulation of ECM deposition, and inhibition of the immune response are effective strategies to reverse liver fibrosis [30, 31]. Activated HSCs around damaged sites recruit macrophages and lymphocytes. Hepatic macrophages, including resident macrophages (Kupffer cells: KCs, resident in liver) and MOMFs (accumulate in the damaged liver site), make great contributions to initiating the hepatic inflammatory response and mediating the development and regeneration of liver fibrosis [34]. KCs recognize damage-associated molecular patterns (DAMPs) or pathogen-associated molecular patterns (PAMPs) released by injured or dead hepatocytes to sense hepatic injury. Activated KCs release pro-inflammatory cytokines and chemokines to attract other immune cells [31, 35]. Hepatic NK and NKT cells play a role in inducing aHSCs apoptosis. They can target natural killer group 2 (NKG2D) receptors on HSCs through caspase 3/8-dependent apoptotic pathways [36] or produce cytokines such as interferon-γ (INF-γ) that affect the signal transducer and activator of the transcription 3 (STAT3) pathway in HSCs [37], thus eliminating aHSCs. In addition, macrophages increase the level of MMP12 and MMP13, thereby facilitating ECM degradation [38, 39]. DCs can also produce MMP9 to disintegrate the ECM [40]. Hepatic DCs are generally involved in immunoregulation by increasing the number of regulatory T (Treg) cells, thereby suppressing liver inflammation to reverse liver fibrosis [41]. Overall, the communication between damaged hepatocytes, immune cells, and HSCs plays an essential role in liver inflammation and influencing the development and regression of liver fibrosis. Recent research on cell-to-cell communication has increasingly focused on EVs, as their secretion and internalization in liver cells affect inflammatory responses during liver fibrogenesis [42].

## Role of immunomodulatory EVs in liver fibrosis

### EVs derived from hepatocytes

Hepatocytes are the primary type of liver cells and are among the initiators of liver inflammation. EVs from hepatocytes function in metabolism, inflammation, and angiogenesis during liver fibrogenesis [19]. Both normal and injured hepatocytes mediate liver immunity by secreting EVs. At the beginning of liver damage, EVs from healthy hepatocytes play a compensatory role in maintaining hepatic inflammatory homeostasis by reducing immune cell aggregation and downregulating inflammatory mediators [10]. In addition, injured hepatocytes are essential sources of EVs involved in liver inflammation; they ‘inform’ immune cells to participate in liver fibrosis and reduce inflammatory infiltration.

In the ALD model, necrotic hepatocytes generate EVs containing various cargo, expediting macrophage infiltration. Previous studies have indicated that hepatocytes exposed to alcohol produce EVs loaded with CD40 ligands (CD40L) or HSP90s to activate macrophages and aggravate liver inflammation in ALD [43, 44]. In addition, alcohol-stimulated hepatocytes transfer EVs enriched with miR-122 to monocytes, which suppresses the heme oxygenase 1 (HO-1) pathway and sensitizing the monocytes to lipopolysaccharide (LPS) while increasing the levels of proinflammatory cytokines [45]. Furthermore, hepatocytes exposed to alcohol upregulate mitochondrial DNA (mtDNA)-enriched EVs due to mitochondrial dysfunction [46]. Alcohol triggers hepatocytes to transfer mtDNA-enriched EVs to augment hepatoxicity and neutrophil infiltration by mediating apoptosis signal-regulating kinase (ASK1) and p38-dependent signaling pathways [47]. These EVs containing mtDNA can stimulate Kupffer cells via activation of Toll-like receptor 9 (TLR9) and upregulate the proinflammatory cytokines, IL-17 and IL-1β [46]. Moreover, in early ALD, alcohol also encourage hepatocytes to release EVs containing mitochondrial double-stranded RNA (mtdsRNA), which can activate both Kupffer cells and γδT cells [48].

EVs secreted by lipotoxic hepatocytes also contribute to the pathogenesis of MASH [49]. In MASH, EV cargo from hepatocytes mediates inflammatory homeostasis through the induction of monocyte/macrophage accumulation, macrophage polarization, and the mediation of proinflammatory cytokines. Palmitate acid-stimulated hepatocytes generate EVs with C-X-C motif chemokine ligand 10 (CXCL10) to cause monocyte/macrophage accumulation via the integrin-β1 signaling pathway. Another study demonstrated that EVs enriched with active integrin β1 (ITGβ1) from hepatocytes can be accepted by monocytes, affecting the adherence of monocytes to hepatic sinusoidal endothelial cells and worsening liver inflammation in a murine MASH model [50]. Palmitate-stimulated hepatocytes also secrete EVs engulfing ceramides and sphingosine-1 phosphate (S1P) through an unfolded protein response sensor in an inositol-requiring protein 1α-dependent manner, which is crucial for ER homeostasis. In addition, EV S1P from hepatocytes induces macrophage accumulation and augments liver inflammation. In further research, lipotoxic hepatocyte-derived EVs loaded with miR-192-5p regulate macrophages through the rapamycin complex 2 (Rictor)/Akt/Fork-head box protein O1 (FoxO1) pathway and polarize them to the M1 phenotype. Another study demonstrated that the influence of lipotoxic hepatocyte-derived EVs on macrophages is mainly polarized [49]. Furthermore, cholesterol negatively affects lysosomal activity in hepatocytes, resulting in the secretion of EVs containing miR-122-5p, which stimulates the release of IL-1 and IL-6 from macrophages and induces M1 polarization [51]. Moreover, lipotoxic hepatocytes also release mtDNA-enriched EVs to upregulate the expression of proinflammatory factors, including TNF-α and IL-1β, in Kupffer cells. EV TNF-related apoptosis-inducing ligand (TRAIL) generated from lipotoxic hepatocytes transfers TRAIL to bone marrow-derived macrophages in the liver and increases the release of IL-1, IL-1β and IL-6 [52]. Moreover, PA-induced hepatocytes secrete miR-107-enriched EVs, which activate HSCs by suppressing dickkopf-1 (DDK1) and Th9 cell differentiation by mediating Forkhead box protein P1 (Foxp1), exacerbating MASH-related liver fibrosis [53].

**Fig. 2:**
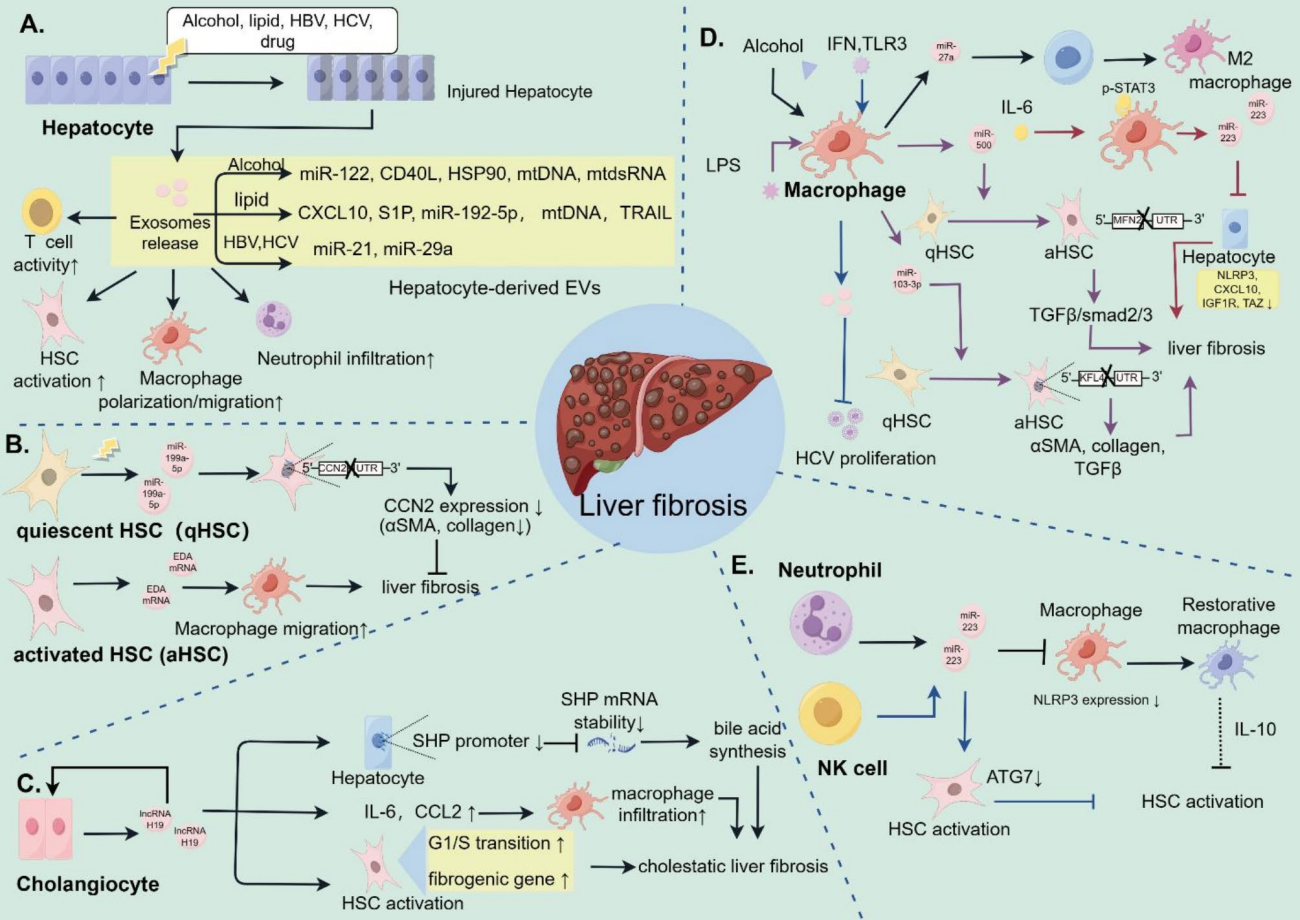
The role of immunomodulatory EVs in the pathogenesis of liver fibrosis. (**A**) EVs derived from injured hepatocytes can activate HSCs, increase T-cell activity, and improve macrophage migration/polarization and neutrophil infiltration. (**B**) EVs originating from HSCs can be received by HSCs and hepatic macrophages. (**C**) Cholangiocyte-derived EVs enriched with lncRNA H19 could mediate the activity of hepatocytes, macrophages, HSCs and cholangiocytes in the development of cholestatic liver fibrosis. (**D**) Macrophages can mediate hepatic inflammation and HSC activation to affect liver fibrosis via EV transfer. (**E**) Both neutrophils and NK cells can secrete miR-223-enriched EVs to regulate macrophages or HSCs

In the context of viral hepatitis, hepatocytes infected by virus [e.g. hepatitis B virus (HBV) and hepatitis C virus (HCV)] likely secrete EVs containing viral RNA or DNA to modulate the liver immune response. EVs enriched in the viral genome are generally phagocytized by macrophages and affect the host immune response [49]. After infection with HBV, the amount of immune-related miRNAs in hepatocyte-derived EVs increase, downregulating the IL-12p35 mRNA level in macrophages and suppressing the immune response in the host [54]. Later, hepatocytes transfected with HBV produce EVs loaded with miR-21 or 29a, inhibiting the production of the proinflammatory cytokine IL-12 [55]. In addition, EVs from HBV-infected hepatocytes act on PD-L1 on macrophages directly, promoting the combination of PD-L1 and PD-1 on T cells and thus inhibiting T cell activation. Furthermore, EVs from HCV-infected hepatocytes can target and activate galectin 9, increasing the levels of T regulatory cells and increasing the rate of apoptosis for HCV-specific T cells [56]. More recently, Dustin A. et al. reported that TGF-β-enriched EVs from HCV-infected hepatocytes act on CD4 + T cells and augment T follicular regulatory cell expansion, subverting the antiviral immune response and promoting the spread of HCV [57]. Additionally, hepatocytes infected by HCV transfer miR-19a via EVs and induce TGF-β-related activation of HSCs, causing excess matrix deposition [58] (Fig. 2A).

### EVs derived from HSCs

HSCs remain in a quiescent state, working as essential storage sites for vitamin A and becoming enriched in retinoid lipid droplets [30]. Activated hepatic stellate cells play a vital role in liver fibrosis, as they accumulate around injured sites and secrete excess ECM [59]. EVs derived from HSCs also function in the development of liver fibrosis. During fibrogenesis, aHSCs secrete exosomes loaded with cellular communication network factor 2 (CCN2), which may accelerate or slow the fibrogenic process [10]. Quiescent HSCs release EVs containing miR-199a-5p, which can be absorbed by aHSCs and suppress their CCN2 expression level by binding with the 3’-UTR (untranslated region) of CCN2 to reduce liver fibrosis [60]. In most cases, EVs are highly involved in the crosstalk between HSCs and immune cells. For example, HSC-secreted EVs in the injured liver increase the expression of IL-6 and TNF-α, exacerbating macrophage migration in human models rather than murine models [61]. In addition, Emillio M. et al. demonstrated that active HSCs exhibit high levels of ectodysplasin-A (EDA) mRNA and deliver EVs loaded with EDA mRNA, stimulating macrophage migration and enhancing the manipulation of the immune response in the liver [62] (Fig. 2B).

### Macrophage- and neutrophil-derived EVs

As the first line of defense against pathogens, hepatic macrophages are important for eliciting an inflammatory response and maintaining systemic homeostasis in the liver, especially bone marrow-derived Ly6C high-expressing macrophages [13, 34]. Macrophages can be classified into two categories: the classical M1 proinflammatory phenotype and the M2 immunoregulatory phenotype. Existing studies indicate that the transformation between M1 and M2 strongly contributes to inflammatory responses and the development of liver fibrosis [34]. As mentioned above, macrophages phagocytose and internalize EVs from damaged hepatocytes. Additionally, macrophages generate EVs that act on targeted cells to participate in the immune response during liver fibrogenesis.

Alcohol exposure leads to the overexpression of miR-155, which targets and reduces the display of lysosomal-associated membrane proteins 1 and 2 (LAMP1 and LAMP2). Under-expressed LAMP1 and LAMP2 increase the number of EVs produced by macrophages [63]. In additional studies, alcohol-stimulated monocytes were shown to secrete EVs enriched with miR-27a, polarizing other monocytes to M2 macrophages in an autocrine manner and mediating the development of ALD [64]. Moreover, LPS-stimulated THP-1 macrophages release EVs containing miR-500 and miR-103-3p, thus increasing HSC activation and eventually proliferation and aggravating liver fibrosis [65, 66]. MiR-500 combines with mitochondrial fusion protein 2 (MFN2) to suppress TGFβ/smad2/3-induced HSC activation [65]. Additionally, miR-103-3p integrates with the 3’UTR of Krüppel-like Factor 4 (KFL4) to increase the expression of fibrotic genes such as α-SMA, TGFβ and Col1a1 [66]. In addition, the level of serum IL-6 is elevated in patients with metabolic dysfunction-associated fatty liver disease (MAFLD), which stimulates the production of miR-223-enriched EVs from macrophages. EVs containing miR-223 can be received by hepatocytes, downregulating the expression of profibrotic transcriptional coactivators with PDZ-binding motifs (TAZ) [67]. Furthermore, the level of miR-690-enriched EVs derived from Kuppffer cells (KCs) significantly decreases during the progression of MASH, which activates de novo lipogenesis (DNL) in hepatocytes and fibrogenic states in HSCs, and inhibits the inflammatory activity of macrophages [68].

As mentioned above, infected hepatocytes transfer viral DNA/RNA to achieve virus propagation. However, in viral hepatitis (HBV or HCV), macrophages undergo polarization or send EVs containing antiviral factors to hepatocytes, which induces an antiviral response. For example, IFN-stimulated macrophages generate EVs to suppress HCV replication [69]. Toll-like receptor 3 (TLR3)-stimulated macrophages release miR-29a-enriched EVs, which can be received by infected hepatocytes and restrain HCV propagation [70] (Fig. 2D).

Even though neutrophils do not persist throughout the entire inflammatory process in liver fibrosis, they contribute to the regression of liver inflammation. During inflammation, neutrophils immediately migrate to injured sites first eliminating pathogens and necrotic cells before they undergo apoptosis [71]. Research has shown that the quantity of neutrophil-derived EVs is positively correlated with the severity of MASH and cirrhosis [72]. Neutrophils produce miR-223-containing EVs and inhibit NOD-like receptor protein 3 (NLRP3) expression in proinflammatory macrophages, inducing a restorative phenotype in macrophages. Moreover, restorative macrophages suppress HSC activation by secreting IL-10 [73]. Previous studies revealed that neutrophils secrete EVs enriched with miR-223, which can be received by hepatocytes and HSCs in MASH. Hepatocytes receive miR-223-enriched EVs by binding apolipoprotein E (APOE) on EVs to low-density lipoprotein receptors on hepatocytes. After that, the expression of TAZ in hepatocytes decreases, indirectly suppressing Hedgehog-dependent signaling in HSCs. However, HSCs that assimilate miR-223-enriched exosomes undergo activation and proliferation via downregulation of GLI family zinc finger 2 (GLI2) and platelet-derived growth factor receptor a/b (PDGFRa/b) [74] (Fig. 2E).

### Natural killer cells and dendritic cell-derived EVs

NK cells play a cytotoxic and cytokine-producing role in the immune response and have been shown to function in liver fibrosis [72]. NK-cell-derived EVs have been confirmed to suppress TGF-β1-triggered HSC activation by coculturing with TGF-β1-educated LX-2 cells when administered to mice with carbon tetrachloride (CCl4)-induced liver fibrosis [75]. In recent studies, NK cells were shown to secrete miR-223-enriched EVs to inhibit TGF-β1-induced HSC activation by downregulating ATG7 (autophagy-related 7) and impeding autophagy [76] (Fig. 2E).

DCs are the predominant antigen-presenting cells (APCs), distinct from other immune cells. They are involved in innate and adaptive immune responses and are involved in supervising the inflammatory microenvironment of liver fibrosis by mediating the activity of macrophages, NK cells, T cells, and HSCs through the TNF-α signaling pathway [77]. Based on accumulating evidence, DC deficiency impairs liver fibrosis, and the presence of a large population of DCs can reverse liver fibrosis [72]; however, the concrete role of DC-derived EVs in liver fibrosis has not been elucidated.

### Other sources of EVs

Some RNA/protein-enriched EVs, encapsulated in the circulatory system after being secreted by parent cells, also mediate the activity of hepatic immune cells. These EVs are derived mainly from hepatocytes and adipose cells. However, research in 2017 demonstrated that adipose tissue is the main source of circulating miRNA-enriched EVs because the depletion of the miRNA-processing enzyme dicer in adipose tissue leads to a significant reduction in the concentration of EV miRNAs in circulation [78]. Previous studies indicated that EVs derived from adipocytes upregulate IL-6 and monocyte chemoattractant protein-1 (MCP-1), which mediate the inflammatory response in the liver [79]. In addition, alcohol induces increased serum EV CYP2E1 (cytochrome P450 family 2 subfamily E member 1) in plasma, which may be generated from hepatocytes and monocytes. These circulating EVs may alter the degree of alcoholic damage to monocytes [80]. In addition, the level of circulating HSP90-enriched EVs is increased in both types of mice subjected to chronic alcohol injection. EVs containing HSP90 play a regulatory role in macrophage polarization [44].

Cholestasis is one of the primary causes of liver inflammation and fibrosis. Under cholestatic conditions, taurocholate acid (TCA) and estrogen induce cholangiocyte secretion of H19-enriched EVs through the activation of the extracellular regulated protein kinase 1/2 (ERK1/2) signaling pathway [81]. In addition, cholangiocyte-derived EV lncRNA H19 can be accepted by cholangiocytes [81], hepatocytes [82], Kupffer cells [83], and HSCs [84]. First, the EV lncRNA H19 promotes cholangiocyte proliferation and accelerates cholestasis in biliary atresia by mediating the spingosine-1-phosphate receptor 2 (S1PR2)/spingosine kinase 2 (SphK2) and let-7/high mobility group AT-hook2 (HMGA2) pathways [81]. Second, the EV lncRNA H19 inhibits the promoter of SHP, which decreases SHP mRNA stability, thus increasing bile acid synthesis and accelerating cholestatic liver fibrosis [81, 82]. Third, excessive production of EV lncRNA H19 increases the levels of the IL-6 and CCL2 to attract infiltrating macrophages and induce monocyte differentiation in a CCL-2/C-C motif chemokine receptor 2 (CCR-2)-dependent manner [83]. Finally, cholangiocyte-derived EV lncH19 can be received by HSCs, which causes them to undergo the G1/S transition and increases HSC proliferation and cholestatic liver fibrosis [84]. Therefore, emerging evidence indicates there is a crucial role of EV lncRNA H19 in cell‒cell communication and hepatic inflammation in the progression of cholestatic liver fibrosis (Fig. 2C).

There are EVs originating from sources outside the liver, including adipose tissue and microbes. For example, parasites can generate EVs enriched with miRNA cargo to achieve parasite‒host communication. Schistosoma japonicum-derived EVs transfer miR-125b to host macrophages and mediate the TLR signaling pathway by acting on tumor-secreted protein S (Pros1) [85]. Furthermore, EVs from Clonrchis sinensis regulate host M1 macrophage polarization via the suppressor of cytokine signaling (Socs1)/C-type lectin domain containing 7 A (Clec7A)-related NF-κB [86].

## The application of EVs in liver fibrosis

### Diagnostic application of EVs in prefibrotic diseases and liver fibrosis

EV builds an excellent bridge for intercellular communication and constitutes a significant “mediator” in the inflammatory microenvironment of liver fibrosis. EVs can be extracted from body fluids such as serum, saliva, and ascites, which can be obtained by the common methods of, filtration, polymer precipitation, immunoaffinity capture, gradient ultracentrifugation, and size exclusion chromatography [24]. Liver biopsy is the gold standard diagnostic method for liver fibrosis, but it has several shortcomings, including invasiveness, bleeding, high price, and a high negative rate [87]. Because these characteristics make detecting liver fibrosis in the early stage challenging, patients may lose a significant prerequisite for curing liver fibrosis—early fibrosis—early diagnosis and estimation of the severity of disease [88]. EVs are employed by transferring various biological substances in vivo, avoiding degradation because of the phospholipid bilayer membrane [89]. In addition, different EV cargos or numbers of EVs are associated with the pathological process and etiology of liver fibrosis [47]. Furthermore, the secretion of EVs under physiological and pathological conditions commonly occurs in both hepatic parenchymal cells (hepatocytes) and nonparenchymal cells, such as immune cells and hepatic stellate cells [90]. Owing to the numerous advantages of EVs and their ability to reflect dynamic information about liver fibrosis, they have become a novel diagnostic method termed “liquid biopsy” and are receiving increasing attention [19].

Persistent hepatic inflammation with the participation of intrahepatic immune cells is the driving force of the development and remission of liver fibrosis [32]. Here, we focused on EV cargos that play a role in the inflammatory microenvironment of liver fibrosis. Currently, EVs have been confirmed as novel and ideal biomarkers for various prefibrotic liver disorders. The concentration and composition of EVs are related to the etiology or severity of liver fibrosis. Previous studies have demonstrated that the circulating EV level is positively correlated with the occurrence of MASH and AH [90, 91]. Additionally, Arianna et al. have demonstrated that total EV count is positively correlated with MASLD, ALD, and autoimmune hepatitis (AIH) in their literature review [92]. Moreover, the amount of total serum EVs is upregulated apparently in bile duct ligation (BDL)–induced liver fibrosis, compared with healthy mice [93]. Thus, total EV count might be an indicator or potential target of liver fibrosis, for instance, the utilization of EV inhibitors. Primarily, miRNAs are a type of EV cargo that emerge in liver injury and participates in hepatic fibrogenesis. In MASH, the levels of EVs containing miR-192-5p, miR-122-5p, and miR-27a from lipotoxic hepatocytes increase and exacerbate MASH [51, 94–97], indicating that they are potential biomarkers that are positively connected with the progression of MASH- and MASH-related liver fibrosis. Moreover, in ALD, EVs containing miR-122, miR-155, and DAMPs, including mitochondrial proteins and DNA, are elevated. miR-122- and DAMP-enriched EVs originate from hepatocytes exposed to alcohol [45, 46], whereas miR-155 and 27a are derived from macrophages/monocytes in ALD [63, 64]. Furthermore, circulating miR-27a has diagnostic potential in HBV-related liver fibrosis and has a positive relationship with HSC activation [98]. Serum EV miR-29-3p, miR-146-5p and miR-27a are upregulated in hepatitis B-induced liver fibrosis [12, 98]. The levels of EV miR-19a, miR-199a, and miR-122 are increased in the serum of patients infected with HCV [56]. HCV-infected hepatocytes produce EVs enriched with miR-19a and stimulate HSCs via a STAT3-dependent pathway, accelerating liver fibrosis. EV miR-19a is also a diagnostic and prognostic biomarker of HCV-related liver fibrosis [58]. However, the levels of EV miR-122-5p and miR-222-3p are decreased after anti-infective treatment, potentially functioning as biomarkers of immune recovery [99]. Furthermore, plasma EV miR-574-5p, miR-500, miR-103-3p, miR-155, miR-29a, and miR-122 are proposed as ideal hallmarks for diagnosing liver fibrosis [12, 100]. Serum exosomal miR-500 and miR-103-3p are higher in the S3-S4 stages of liver fibrosis compared to the early phases, indicating their potential to predict the progression of liver fibrosis [64, 65]. Furthermore, miR-92a and miR-146a-5p in serum EVs increase significantly in advanced liver fibrosis compared with early or mild stages and positively correlate with the severity of liver fibrosis [95]. Serum enclosed-miR-155 EVs also increase apparently in Child-Paugh C and is positively correlated with reduced survival time after liver transplantation [51]. Thus, miR-155 in EVs might be useful for detecting advanced liver fibrosis and predicting prognosis of cirrhosis. In addition to microRNAs, circulating EV lncRNAs and circRNAs also have great value in monitoring the progression of liver fibrosis. Previous studies have indicated that EV circ-death inducer-obliterator 1 (circ-DIDO1) is positively correlated with liver fibrosis because of its ability to suppress HSC activation via the miR-141-3p/PTEN/AKT pathway [101]. In addition, the EV lncRNA H19 can function as a hallmark of cholestatic liver fibrosis [81]. Additionally, as injured hepatocytes are the main force of EV production in liver inflammation, the level of hepatocyte-derived EVs has a significant positive connection with the severity of liver fibrosis, which is assessed by the Child‒Pugh score [102]. Moreover, several serum EV proteins can also predict the severity of liver fibrosis. For example, CYP2E1-enriched EVs can serve as diagnostic biomarkers of alcohol- and acetaminophen-induced liver diseases. EV cytochrome P450 family 3 subfamily A member 4 (CYP3A4) is also positively related to drug-induced liver diseases, such as rifampin, herbal products and, phenobarbital [80]. The increased level of serum CD206-enriched EVs derived from macrophages is associated with a diagnosis of alcoholic liver fibrosis [103]. Primary biliary cholangitis (PBC) and primary sclerosing cholangitis (PSC) are chronic cholestatic liver diseases influenced by autoimmunity [104]. The level of serum EVs containing miR-451a and miR-642a-3p are upregulated in patients with PBC [105]. Additionally, emerging evidence indicates the amount of cholangiocyte-derived EVs enriched with lncRNA H19 increases in mice and humans with PBC/PSC, suggesting it could be a potential diagnostic marker for cholestatic liver fibrosis [84].

Non-invasive fibrosis tests (NITs) are commonly used in clinical diagnosis for assessing and staging liver fibrosis, which includes blood-based biomarkers, elastography, sonographic elastography techniques, and MR elastography. Various combinations of blood biomarkers can assess liver fibrosis from different perspectives. For example, the AST-Platelet Ratio Index (APRI) consists of aspartate aminotransferase (AST) and platelets detection, which has 50-60% indeterminate cases and limited prognostic ability [106]. Combining serum EV cargo with other NITs to monitor the progression and severity of liver fibrosis shows significant value in early diagnosis and enhancing therapeutic efficacy. For instance, miR-122 in circulating EVs has increased accuracy when cooperating with FIB-4 and transient elastography. The use of serum EV cargos to monitor the progression and severity of liver fibrosis is highly valuable for early diagnosis and increased therapeutic efficacy. However, serum EVs seem to be are more suitable as an auxiliary method for diagnosing liver fibrosis and guiding therapy. Given that the false-positive and negative rates of EVs in diagnosing liver fibrosis are unclear, increasing the sensitivity and specificity of diagnostic EVs in clinical trials is urgently needed for future investigations [107]. Thus, serum EVs have great potential as an auxiliary method for monitoring the development of liver fibrosis and guiding more precise therapy. However, factors such as aging, gender, and etiology should be considered to draw more accurate and comprehensive conclusions. (Table 1)

**Table 1:** Biomarker potential of immunomodulatory EVs in liver fibrosis

Diseases	Source	Effective component	Expression	Potential function		Reference
MAFLD	Hepatocyte	miR-192-5p	Upregulation	Induce the polarization of M1 macrophage and indicate the progression of MAFLD-related liver fibrosis.		[108]
MAFLD	Hepatocyte	miR-122-5p	Upregulation	Lead to M1 polarization and suggest the severity of MAFLD.		[51]
MAFLD	Hepatocyte	miR-27a	Upregulation	Suppress mitophagy and accelerate MAFLD-related fibrosis.		[94–96]
ALD	Hepatocyte	miR-122	Upregulation	Reprogram monocytes and indicate liver fibrosis.		[45]
ALD	Macrophage/monocyte	miR-155	Upregulation	Predict ALD and mediate autophagy and lysosome function in ALD.		[63]
ALD	Macrophage/monocyte	miR-27a	Upregulation	Induce monocyte differentiation to M2 macrophage.		[64]
ALD	Hepatocyte	DAMPs (mitochondrial proteins and DNA)	Upregulation	Mediate the activation of hepatic macrophages and work as biomarker of alcoholic liver injury.		[46]
ALD	Serum	CYP2E1	Upregulation	Diagnosis of alcoholic or acetaminophen-induced diseases		[80]
ALD	Serum	CYP3A4	Upregulation	Diagnosis of drug-induced liver diseases		[80]
ALD	Serum	CD206	Upregulation	Diagnosis of alcoholic liver fibrosis		[103]
HBV	Serum	miR-29-3p	Upregulation	Diagnosis of advanced chronic hepatitis B-induced liver fibrosis.		[12]
HBV	Serum	miR-146-5p	Upregulation	Diagnosis of advanced chronic hepatitis B-induced liver fibrosis.		[12]
HBV	Serum	miR-27a	Upregulation	Indicate HSC activation and hepatitis B virus-induced liver fibrosis.		[98]
HCV	hepatocyte	miR-19a	Upregulation	Activate HSC and function as a diagnostic and prognostic biomarker of HCV-related liver fibrosis.		[58]
HCV	Serum	miR-199a	Upregulation	Indicate HCV infection.		[56]
HCV	Serum	miR-122	Upregulation	Indicate HCV infection.		[56]
HCV	Serum	miR-122-5p	Downregulation	Indicate immune recovery after anti-infective treatment		[99]
HCV	Serum	miR-222-3p	Downregulation	Indicate immune recovery after anti-infective treatment		[99]
PBC	Serum	miR-451a and miR-642a-3p	Upregulation	Increase with the severity of PBC.		[105]
Liver fibrosis	Serum	miR-103-3p	Upregulation	Predict liver fibrosis in S3-S4 stages		[72]
Liver fibrosis	Serum	miR-500	Upregulation	Predict liver fibrosis in S3-S4 stages		[65]
Liver fibrosis	Serum	Cir-DIDO1	Upregulation	Diagnosis of liver fibrosis.		[101]
Liver fibrosis	Serum	miR-29	Downregulation	Low circulating miR-29 indicates end-stage liver fibrosis.		[100]
Liver fibrosis	Serum	miR-92a and miR-146a-5p	Upregulation	Increase with the progression of liver fibrosis.		[109]
Liver fibrosis	Serum	miR-155	Upregulation	Increase in Child-Paugh C of liver fibrosis.		[51]
Liver firbosis	Serum	LncRNA-H19	Upregulation	Diagnosis of cholestatic liver fibrosis		[81]

### Therapeutic application of EVs in liver fibrosis

#### Natural EV therapy in the inflammatory microenvironment of liver fibrosis

EVs are absorbed by target cells via membrane fusion, pinocytosis, and endocytosis, thereby transmitting signals through EV surface proteins or through the cargo they take [110]. Owing to their good transport efficiency, low immunogenicity, biocompatibility, and low toxicity, the therapeutic value of EVs has been discussed in depth [110, 111]. The administration of natural EVs derived from healthy people can transfer effective constituents to target cells, mediating inflammatory reactions in the pathogenesis of liver fibrosis and partly slowing its progression [53].

First, some immune cells produce EVs to repress liver fibrosis. It has been confirmed that injecting EVs secreted by DCs and NKs relieves the inflammatory response in the liver [10]. Administered EVs derived from bone marrow-derived DCs can keep Tregs and Th17 cells in equilibrium, increasing liver function in mice with ischemia/reperfusion injury [112]. In addition, the injection of EVs produced by immature DCs with donor-specific Tregs is beneficial for attenuating rejective reactions after liver allografts and extending the lifespan of liver allografts in rats [113]. Moreover, Wang et al. reported that NK-derived EVs inhibit TGF-β1-activated HSCs and increase liver function in CCl4-induced liver fibrosis in mice [75]. As mentioned above, miR-223 is abundant EV derived from NK cells and neutrophils and may play a role in inactivating HSCs [114]. Macrophage-derived EVs impact immunomodulation by delivering antigens to other immune cells and prolonging the active status of their recipient immune cells [53]. Moreover, EV miR-411-5p from M2 macrophages suppresses HSC activation by targeting calmodulin-regulated spectrin-associated protein 1 (CAMSAP1), which is one of the plausible aims of MASH treatment [115].

Second, the potential therapeutic effects of MSC-derived EVs (MSC-EXos) in liver diseases have been confirmed [116]. MCSs are pluripotent stem cells that originate from early embryonic development, are widely used for regulating inflammation and are easily isolated from diverse tissues, including bone marrow, muscles, the umbilical cord, adipose tissue, and tendons. Bone marrow MSCs (BM-MSCs), menstrual blood MSCs (Men-SCs), umbilical cord MSCs (UC-MSCs), and adipose tissue MCSs (AD-MSCs) have the potential to differentiate into hepatocyte-like cells. Previous studies revealed that MSCs assist with tissue regeneration by secreting paracrine factors packaged in EVs rather than cell replacement. Therefore, MSC-EVs have become an ideal cell-free therapy for treating liver fibrosis and have attracted increasing attention because of their promising immunosuppressive effects and ability to induce EV production [117–119]. MSC-EVs restrain cirrhosis by inhibiting hepatocyte apoptosis, suppressing HSC activation and proliferation, decreasing collagen deposition, reducing EMT, and increasing the inflammatory response in the liver [117]. Rong et al. reported that human bone marrow MSC-Exos target the Wnt/β-catenin pathway, repress HSC activation and reduce inflammation in mice with CCl4-induced liver fibrosis [120]. In addition, Zhang et al. demonstrated that human adipose MSCs ameliorate liver fibrosis by restraining the PI3K/AKT/mTOR signaling pathway to suppress HSC activation and increase the metabolic rate of choline-phosphatidylcholine in a dose-dependent manner [121]. Moreover, MSC-EVs promote the function of Treg cells and upregulate a series of cytokines, thereby playing an immunosuppressive role in a concanavalin A-induced liver injury murine model [122]. Subsequently, research on therapeutic cargo packaged by MSC-EVs emerged. EVs derived from human tonsil-derived MSCs (T-MSCs) transfer miR-486 to inactivate HSCs by inhibiting the hedgehog signaling pathway, playing an antifibrotic role in CCl4-induced liver fibrosis [123]. AD-MSCs produce EVs loaded with miR-150-5p to reduce the level of the CXC chemokine-ligand-1 (CXCL1) in CCl4-induced liver fibrosis [124]. Additionally, Chen et al. demonstrated that EVs derived from human placental MSCs (hpMSCs) could suppress Th17 differentiation by inhibiting IκBζ, thereby ameliorating PSC-related liver fibrosis in multidrug resistance gene 2 knockout (Mdr2-/-) mice—a genetic model of PSC that mimics the canalicular phospholipid flippase deficiency seen in patients, leading to toxic bile acid accumulation and hepatocyte injury [125]. 

**Fig. 3:**
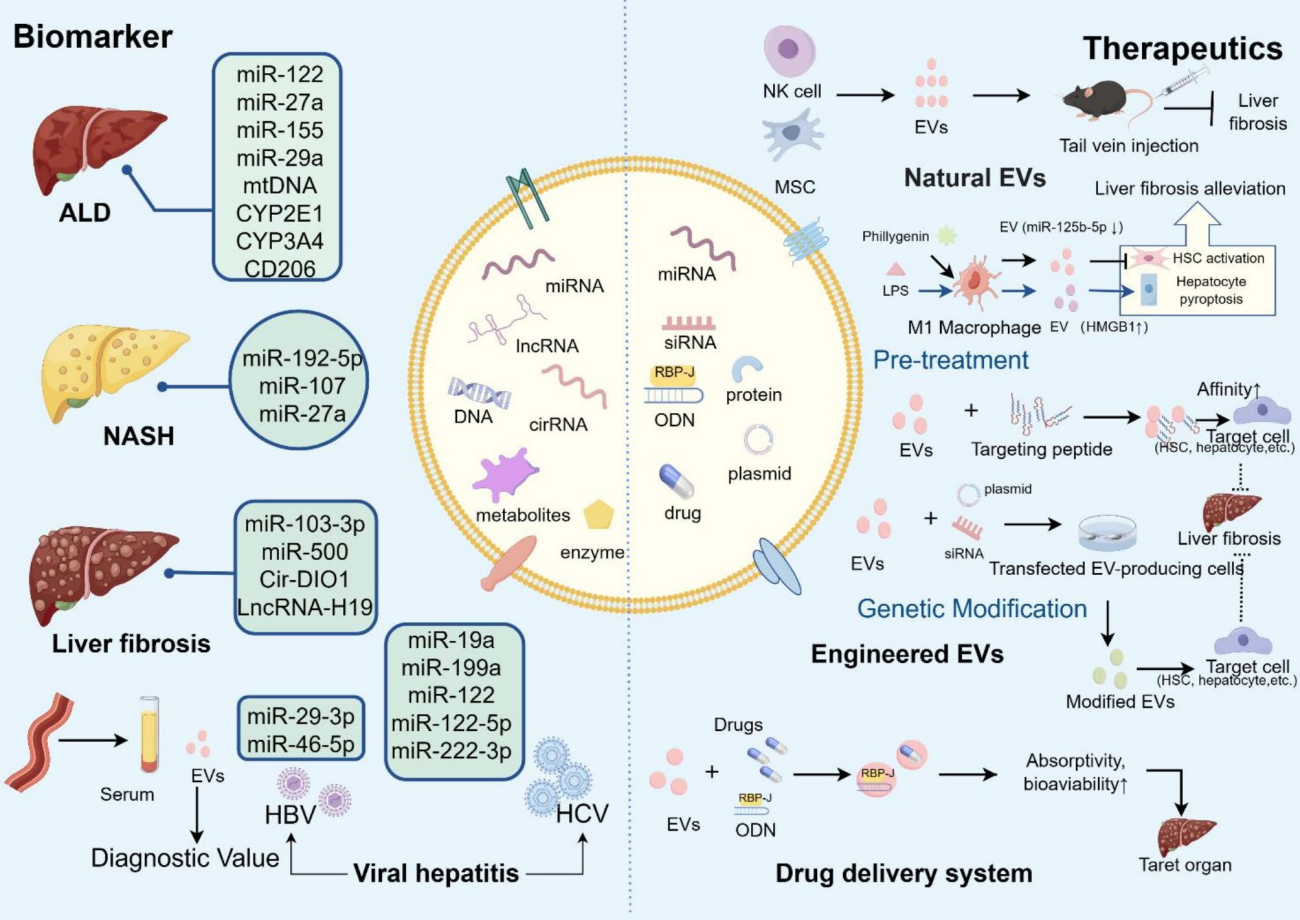
Clinical application of immunomodulatory EVs in liver fibrosis. The cargo of immunomodulatory EVs originate from parenchymal/nonparenchymal cells and is closely associated with the pathological process of liver fibrosis, revealing the diagnostic potential of EVs in predicting the etiology and severity of liver fibrosis. The therapeutic value of natural and engineered EVs has been demonstrated in previous studies. EVs also provide antifibrotic drugs as effective delivery platforms to increase their absorptivity in vivo

Overall, natural EV therapy is considered a potential candidate for alleviating liver fibrosis. Currently, several predominant obstacles to naive EV therapy need to be addressed, including phagocytosis by the mononuclear-phagocyte system, tedious methods for extracting and purifying natural EVs, optimal administration and dosage strategies, and a short half-life [126–128]. The safety and efficacy of these compounds, as well as their antifibrotic mechanisms, urgently require validation in further scientific experiments and clinical trials (Fig. 3).

**Table 2:** Therapeutic potential of immunomodulatory EVs in liver fibrosis

Source	Modification/Treatment	Effective components	Expression	Administration route	Therapeutic implication	Reference
M1 macrophage	Phillygenin	miR-125b-5p	Downregulation	-	Inactivate HSCs and may suppress liver fibrosis	[129]
Macrophage	LPS	HMGB1	Upregulation	-	Induce hepatocyte pyroptosis and show therapeutic value in acute liver injury.	[130]
Macrophage	Myeloid-specific IL-6	miR-223	Upregulation	Tail vein injection	Suppress pro-fibrotic gene expression in hepatocytes and controlling MAFLD-related liver fibrosis.	[67]
AMSC	Transfection	miR-122	Upregulation	Intravenous route	Dampen HSC activation and proliferation to alleviate CCl4-induced liver fibrosis.	[131]
ADSC	Transfection	miR-181-5p	Upregulation	Intrasplenic route	Initiate autophagy in HST cells and CCl4-induced liver fibrosis.	[132]
MSC	Transfection	circDIO1	Upregulation	-	Result in apoptosis, cell cycle arrest in HSCs.	[101]
293T	Electroporation	RBP-J ODNs	Upregulation	Tail vein injection	Ameliorate CCl4- and BDL-induced liver fibrosis	[133]
ASC	Electroporation	OPN	Downregulation	Intravenous route	Inhibit liver fibrosis through TGF-β signaling pathway.	[134]
LX-2	Electroporation	Cas9 RNP complex	-	Tail vein injection	Target CcnE1 precisely and suppress the initiation of liver fibrosis.	[135]
hpMSC	-	-	-	Tail vein injection	Alleviate PSC-related liver fibrosis through mediating Th17-induced microenvironment.	[125]

#### Modified EV therapy in the inflammatory microenvironment of liver fibrosis

Compared with other synthetic nanocarriers such as liposomes, nanoparticles, and microspheres, EVs are promising drug delivery tools for various diseases due to their biocompatibility, physiological stability, and biological barrier penetrative stability [136]. Therapeutic agents, including nucleic acids, drugs, metabolites, and enzymes packaged in EVs, exhibit delayed disintegration and high absorbability [117]. Decoding EV surface proteins and cargo by engineering technologies can significantly increase their targeting sensitivity and therapeutic efficacy [137]. EV surface protein/ligand modifications can be achieved by genetic engineering or direct preconditioning, increasing the specificity of their ability to target specific cell types [137]. A sequence of a target protein/peptide is genetically fused with one of the EV membrane proteins [such as Lysosome-associated membrane glycoprotein (LAMP-2B) and platelet-derived growth factor receptor (PDGFR)] via genetic engineering [118]. Surface-engineered EVs are generated by transfecting a plasmid encoding a fusion gene into parent cells [15, 136]. Then, transfected parent cells produce modified EVs expressing targeting ligands on their surface and ensure their promising localization in targeted tissues/organs [118, 137]. Previous studies have demonstrated that acidified Lamp2b-HuR-fused protein enables RNA targets to be degraded by lysosomes in recipient cells and significantly alleviates liver fibrosis [138]. Nonetheless, modified EV surface proteins may be disintegrated by cellular proteases. Later, scientists added glycosylation motifs to peptide-Lamp2b fusion proteins to avoid peptide degradation [139]. In addition, EVs, which are distinct from live cells, can utilize covalent or noncovalent modifications to alter the properties of surface proteins [118, 137]. However, both chemical manipulation and transfection of EV-producing cells with plasmids can partially impair the normal function of modified surface proteins, which still needs further study [15]. Currently, surface-modified EVs show great potential in targeted therapeutic delivery systems for the treatment of cancers, neurodegenerative diseases, and cardiovascular diseases. In addition to previous guidelines, their application in antifibrotic therapy deserves further investigation [136]. Lin Y. et al. proposed that fusing the surface proteins of therapeutic EVs with peptides targeted to HSCs could regulate HSC activation and potentially reverse liver fibrosis [136].

Therapeutic bioactive molecules, such as nucleic acids, proteins, metabolites, and enzymes can be inserted into EVs via pretreatment and genetic engineering, which induces the expression of promising RNAs, proteins, or receptors in these EVs, resulting in additional functions [14]. Preprocessing EVs under specific conditions to alter their cargo composition common in modified EV therapy for liver fibrosis. For example, pretreating M1 macrophages with phillygenin downregulates their secretion of miR-125b-5p, reversing the activation of HSCs and attenuating liver fibrosis by targeting StAR-related lipid transfer domain-containing 13 (Stard13). Although the ability of phillygenin to suppress macrophage polarization and HSC activation has been identified in vitro, its efficiency and potential adverse effects in animal models and clinical trials remain unclear [129]. In addition, LPS-induced macrophages produce EV containing high mobility group box protein 1 (HMGB1) to initiate hepatocyte pyroptosis via stimulation of NOD-like receptor thermal protein domain-associated protein 3 (NLRP3) inflammasomes, providing potential diagnostic and therapeutic tools for acute liver injury [130]. In the MAFLD murine model, myeloid-specific IL-6 increases the level of EV miR-223 originating from macrophages by stimulating the expression of genes related to EV biogenesis in macrophages. MiR-223-enriched EVs are received by hepatocytes, where they target and downregulate profibrotic gene expression [67]. Although pretreating EV-producing cells with inductive agents can alter genetic information in EVs and partly enhance the efficacy of exosome therapy for liver fibrosis, standards for assessing the dosage and duration of these induction agents need to be established for pretreating therapeutic EVs. Genetic engineering techniques, such as gene addition, gene silencing, and gene editing, are used to introduce extrinsic materials into EVs via co-transfection, electroporation, and transfection [131, 136, 140]. Genetic addition is commonly achieved by transfecting target RNAs into EV-producing cells, such as MSCs. For example, transferring premiR-122 via a lentivirus to create miR-122-modified AMSCs can increase the serum concentration of EV miR-122 to dampen fibrogenesis by inhibiting HSC proliferation and collagen deposition [131]. Similarly, overexpressing miR-181-5p in ADSCs via transfection can alternatively generate miR-181-5p-modified EVs that are transferred to injured hepatocytes alternatively, which impede autophagy by blocking the signal transducer and activator of transcription 3 (STAT3)/ (B-cell lymphoma 2) Bcl-2/Beclin 1-dependent pathway [132]. In addition, MSC-Exos containing excess circDIO1 were found to restrain HSC activation by obstructing the miR-141-3p/PTEN/AKT pathway in vitro; however, further fibrotic animal experiments are needed [101]. In addition to gene addition, gene silencing-modified EVs also contribute to antifibrotic therapy for liver fibrosis. For example, osteopontin (OPN) has been confirmed as a critical mediator of liver fibrosis. Engulfing OPN-small interference RNA (siRNA) into EVs isolated from ADSCs and injecting them into CCl4-induced liver fibrosis models can reverse HSC activation and ECM deposition, thereby mitigating liver fibrosis and increasing liver function [134]. Furthermore, the use of clustered regularly interspaced short palindromic repeats (CRISPR) and CRISPR-associated protein 9 (Cas9) technology provides convenience for editing EVs in the treatment of liver fibrosis. EVs also offer an efficient and safe delivery mode to Cas9 ribonucleoprotein (RNP)--- a large component of the CRISPR-Cas9 system [135]. CRISPR/dCas9-VP64 plasmids can be loaded into AML12 cells and transferred to HSCs in an EV manner [141]. Wan et al. encapsulated Cas9 RNP complexes into EVs extracted from the LX-2 cell line via electroporation. Systemic administration of EVs loaded with Cas9 RNPs enable gene therapy for liver fibrosis via significant downregulation of cyclin E1 (CcnE1) [135]. Cas9 RNPs represent a novel method for gene editing for precise and tissue-specific therapy of liver fibrosis, and EVs provide a suitable delivery tool for RNPs. In brief, artificial modification enhances the targeting ability of natural EVs by increasing spatial specificity through the anchoring of targeting ligands on their surfaces and improving therapeutic efficacy by increasing the load of therapeutic agents [142] Additionally, the extended circulation time and well-established degradation mechanisms of surface-modified EVs enhance their biological stability in vivo. However, surface-modified EVs may fail to achieve specific organotropism due to the inactivation of artificially added surface ligands [118]. Although modified EVs show considerable potential in therapeutic applications for liver fibrosis, regulatory and safety concerns in their production and use still require thorough investigation. (Table 2)

Some drugs can be engulfed by EVs to delay clearance and increase transport efficiency in vivo. The development of therapeutic agents with EVs frequently involves manipulations such as electroporation, lipofection, sonication and coincubation, extrusion, freezing, and saponin-mediated permeabilization [143, 144]. Milk-derived EVs taken with forsythiaside A (FA) target CD44, controlling NLRP3-mediated pyroptosis and dampening liver fibrosis. FA, a useful drug for treating liver fibrosis, has poor absorptivity, permeability, retention, and bioavailability. Using EVs as vehicles can solve these problems [145]. Moreover, luteolin can be loaded in BMSC-Exos to cope with liver fibrosis [145]. The super repressor IκBα-loaded EVs downregulate fibrosis-related genes in HSCs and attenuate macrophage and neutrophil accumulation, suppressing ALD-related liver fibrosis. EVs encapsulate the super repressor IκBα via a novel EV-modifying method named ‘EVs for protein loading via optically reversible protein-protein interactions’ (EXPLOR) [146]. Additionally, loading RBJ-decoy oligodeoxynucleotides into EVs derived from 293T cells via electroporation can target hepatic macrophages, inhibiting hepatic inflammation and liver fibrosis in mice by suppressing the Notch signaling pathway [133]. Additionally, traditional Chinese medicines, including monomeric active ingredients and compound preparations, can be transported by EVs [18]. Currently, emerging studies have proposed novel EV-liposome hybridization nanodrug delivery systems, such as EV-liposome hybrids, which can transfer clodronate-nintedanib (a combined antifibrosis drug) to Kupffer cells, restraining their activity and reducing liver fibrosis [147]. However, the rate of drug loading in EVs depends on the hydrophobicity and the loading method of the drug [148]. Therefore, choosing an applicable drug-loading method based on the properties of drugs is important for drug-loaded EVs. Enveloping therapeutic agents within EVs often involves techniques such as electroporation, lipofection, sonication, co-incubation, extrusion, freeze-thaw cycles, and saponin-mediated permeabilization [143, 144]. However, extrusion, which is commonly used to load drug cargo into EVs by utilizing an extruder, disrupts the integrity of the EV membrane and results in low encapsulation efficiency. Electroporation achieves transient permeabilization of the EV membrane by applying a short high-voltage pulse. Despite being relatively mature and widely used, electroporation still disturbs the integrity of EV membranes and damages protein structures, reducing loading efficiency and influencing EV biogenesis [18]. Developing efficient drug-loading techniques that efficiently encapsulate cargo into EVs without disruption of EV membrane proteins remains a significant challenge in the field. Furthermore, encapsulating the exogenous cargo into EVs might interact with endogenous cargo of EV and whether this causes off-target effects still requires further exploration [149]. (Fig. 3).

According to the above information, it is reasonable to conclude that EV is a novel and ideal diagnostic and therapeutic method for liver fibrosis treatment.

## Discussion and perspectives

In the past decade, the significant value of EVs in mediating the inflammatory response in liver fibrosis has been confirmed. EVs derived from different cells interact with each other through their specific cargos, exerting beneficial or detrimental effects on the inflammatory microenvironment of liver fibrosis. Furthermore, donor cells secrete EVs that carry varying quantities and components of loads under physiological and pathological conditions. Thus, assessing the level, content, and origin of exosomes via lipid biopsy has emerged as a novel cell-free, noninvasive and accessible method for estimating the etiology and severity of liver fibrosis. Previous studies have revealed correlations between various EV cargos and the etiology of liver fibrosis. However, only a few studies have reported that specific EV cargos are expressed at different stages of liver fibrosis, and additional experimental data along with more precise classification of the fibrotic stage are needed. In this study, we carefully reviewed the EV biomarkers involved in inflammatory regulation based on disease type. However, the diagnostic use of EVs in liver fibrosis is still in its infancy. The lack of standardized methods for isolation and characterization influences the sample preparation, and reliability of EV-based biomarkers. The heterogeneity of EVs varies between different individuals based on age, gender, physiological, and pathological conditions. Thus, establishing standardized parameters to distinguish healthy controls from patients can be a great challenge [150]. Moreover, the sensitivity and specificity of diagnostic EVs for detecting or predicting liver fibrosis requires a large cohort of patients in clinic tests, which is essential for precise diagnosis [149].

In addition to their use in diagnostics, EVs are applied in drug delivery, EV therapy, and infectious disease vaccines. When delivered to target cells, EVs regulate the activity of recipient cells through various signaling pathways. The mechanisms of EV therapy in liver fibrosis primarily involves the inhibition of HSC activation and alleviation of hepatic inflammation. The utilization of natural and modified EVs in liver fibrosis exhibit promising therapeutic effects. EV therapy provides a novel direction for drug delivery and genetic therapy for liver fibrosis with significant advantages in protecting therapeutic agents from degradation and delivering them to target cells with high selectivity and safety. However, well studies on EV therapy for liver fibrosis are abundant at the stage of animal experiments, clinical trails are still lacking. According to *ClinicalTrials.gov*, Kaveh Baghaei conducted a study to identify the therapeutic safety and efficacy of EVs derived from umbilical cord-derived MSCs in decompensated liver fibrosis. Additionally, EV-based vaccines have been recognized as potential methods to address cancers and infectious diseases, such as DC-derived EVs isolated from cancer patients [14, 24]. Nevertheless, the development of immunotherapeutic vaccines for liver fibrosis or chronic liver diseases should be further explored.

In addition, the administration route, dosage, and adverse effects of EVs must be evaluated in clinical trials to broaden their clinical utilization. Several aspects of therapeutic EVs from immune cells in liver fibrosis remain uncertain and require optimization: (a) stable storage and transportation of EVs, (b) examination of EV production quality and control, (c) more precise and effective instruments for large-scale production and EV purification [53], (d) the criterion for choosing the proper administration route and accurate dose, (e) the application of EV mimics, and (f) stable inductive condition for immune cells in vitro. A better understanding of the function and mechanism of EVs in inflammatory microenvironments may lead to the development of novel cell-free therapies for treating liver fibrosis.

## Conclusions

EVs provide a vital and efficient platform for cell‒cell communication in the inflammatory microenvironment of liver fibrosis. The prominent advantages of these methods, including their noninvasiveness and cell-free nature, high specificity and low toxicity, make them suitable for drug delivery and genetic therapy for liver fibrosis. This review summarizes current developments in the mechanism and application of immunomodulatory EVs involved in the pathogenesis of liver fibrosis and provides novel and comprehensive insights into the precise diagnosis and personalized treatment of liver fibrosis.

## Data Availability

No datasets were generated or analysed during the current study.

## Abbreviations

AD-MSCs: Adipose tissue MCSs
AD-MSCs: Adipose tissue MCSs
aHSCs: Activated hepatic stellate cells
AIH: Autoimmune hepatitis
ALD: Alcoholic liver diseases
Alix: ALG-2 interacting protein X
ALIX: ALG-2 interacting protein X
APCs: Antigen-presenting cells
APOE: Apolipoprotein E
APRI: AST-Platelet Ratio Index
ASK1: Apoptosis signal-regulating kinase
AST: Aminotransferase
BDL: Bile duct ligation
BM-MSCs: Bone marrow MSCs
BM-MSCs: Bone marrow MSCs
CAMSAP1: Calmodulin-regulated spectrin-associated protein 1
Cas9: CRISPR-associated protein 9
CCL2: Chemokine ligand
CCl4: Carbon tetrachloride
CCN2: Cellular communication network factor 2
CcnE1: Cyclin E1
CCR-2: Chemokine receptor 2
CD40L: CD40 ligands
circ-DIDO1: Circ-death inducer-obliterator 1
Clec7A: Containing 7 A
CRISPR: Clustered regularly interspaced short palindromic repeats
CXCL10: Chemokine ligand 10
CXCL1: Chemokine-ligand-1
CXCR4: C-X-C motif chemokine receptor 4
CYP2E1: Cytochrome P450 family 2 subfamily E member 1
CYP3A4: Cytochrome P450 family 3 subfamily A member 4
DAMPs: Damge-associated molecular pattern
DAMPs: Mitochondrial proteins
DCs: Dendritic cells
DDK1: Dickkopf-1
DNL: De novo lipogenesis
ECM: Excessive extracellular matrix
EDA: Ectodysplasin-A
ERK1/2: Extracellular regulated protein kinase 1/2
ESCRT: Endosomal-sorting complex is required for transport
ESEs: Early sorting endosomes
EVs: Extracellular vesicles
FoxO1: Fork-head box protein O1
Foxp1: Forkhead box protein P1
GLI2: GLI family zinc finger 2
HBV: Hepatitis B virus
HCC: Hepatocellular carcinoma
HCV: Hepatitis C virus
HMGA2: High mobility group AT-hook2
HMGB1: High mobility group box protein 1
HO-1: Heme oxygenase 1
hpMSCs: Human placental MSCs
HSCs: Hepatic stellate cells
ILVs: Intraluminal vesicles
INF-γ: Interferon-γ
ITGβ1: Integrin β1
KCs: Kuppffer cells
KFL4: Krüppel-like Factor 4
LAMP-2B: Lysosome-associated membrane glycoprotein
LAMP1: Lysosomal-associated membrane proteins 1
LAMP2: Lysosomal-associated membrane proteins 2
LESs: Late endosomes
LPS: Lipopolysaccharide
LSEs: Late-sorting endosomes
MAFLD: Metabolic dysfunction-associated fatty liver disease
MASH: Metabolic dysfunction-associated steatohepatitis
MCP-1: Monocyte chemoattractant protein-1
Mdr2-/-: Multidrug resistance gene 2 knockout
Men-SCs: Menstrual blood MSCs
Men-SCs: MSCs
MFN2: Mitochondrial fusion protein 2
miRNAs: MicroRNAs
MoMFs: Monocyte-derived macrophages
MSC-EXos: MSC-derived EVs
MSCs: Mesenchymal stem cells
mtDNA: Mitochondrial DNA
mtdsRNA: Mitochondrial double-stranded RNA
MVBs: Multivesicular bodies
NITs: Non-invasive fibrosis tests
NK cell: Natural killer cell
NKG2D: Natural killer group 2
NKT cell: Natural killer T cell
NLRP3: NOD-like receptor thermal protein domain-associated protein 3
OPN: Osteopontin
PAMPs: Pathogen-associated molecular patterns
PBC: Primary biliary cholangitis
PDGFR: Platelet-derived growth factor receptor
PDGFRa/b: Platelet-derived growth factor receptor a/b
Pros1: Protein S
PSC: Primary sclerosing cholangitis
RNP: Ribonucleoprotein
ROS: Reactive oxygen species
S1P: Sphingosine-1 phosphate
S1PR2: Spingosine-1-phosphate receptor 2
SiRNA: Small interference RNA
SNARE: Soluble N-ethylmaleimide-sensitive factor attachment protein receptor
Socs1: Suppressor of cytokine signaling
SphK2: Spingosine kinase 2
STAT3: Signal transducer and activator of transcription 3
T-MSCs: Tonsil-derived MSCs
TCA: Taurocholate acid
TGF-β: Transforming growth factor-β
TLR3: Toll-like receptor 3
TLR9: Toll-like receptor 9
TNF-α: Tumor necrosis factor-α
TRAIL: TNF-related apoptosis-inducing ligand
TSG101: Tumor Susceptibility Gene 101
UC-MSCs: Umbilical cord MSCs
